# Postoperative Atrial Arrhythmias After Lung Transplantation: A Single Center Analysis of Risk Factors, Management, and Outcomes

**DOI:** 10.1111/ctr.70457

**Published:** 2026-01-30

**Authors:** Daniel M. Chen, Ambalavanan Arunachalam, Graham Peigh, Bradley P. Knight, Chitaru Kurihara, Mrinalini Venkata Subramani, Catherine Myers, Alan Betensley, Krishnan Warrior, Bradford C. Bemiss, Amanda Kamar, Mohamed Al‐Kazaz

**Affiliations:** ^1^ Feinberg School of Medicine Northwestern University Chicago Illinois USA

**Keywords:** anticoagulation, arrhythmia‐related hospitalization, beta‐blocker, late atrial arrhythmia, mortality, pulmonary arterial hypertension, stroke

## Abstract

**Background:**

Postoperative atrial arrhythmia (POAA) is common after lung transplant, but data on its implications and management are limited. This study assessed POAA incidence and timing, its association with mortality and rehospitalizations, and outcomes related to postoperative beta‐blocker use.

**Methods:**

We retrospectively studied 233 adult lung transplant patients at Northwestern Memorial Hospital (2014–2024) without prior atrial arrhythmia. POAA, defined as atrial fibrillation or flutter, was confirmed by ECG or ambulatory monitoring. Multivariable logistic and Cox regression models evaluated predictors and outcomes, adjusting for covariates.

**Results:**

POAA occurred in 69/233 patients (29.6%) and was associated with higher mortality (HR3.09, 95%CI [1.57–6.08]). Among these, 18/69 (26.1%) developed POAA after the index hospitalization at median 216 days post‐transplant, also associated with higher mortality (HR4.37, 95%CI [1.90–10.06]). Additionally, 10/69 (14.5%) required emergency visits or hospitalization specifically for arrhythmia management, and 66.6% underwent additional rhythm control (2.9% cardioversion, 46.4% anti‐arrhythmic drug, 17.4% both). Postoperative beta‐blocker use was associated with 73% less POAA (HR0.27, 95%CI [0.09–0.80]).

**Conclusion:**

POAA was common and clinically significant after lung transplant, associated with substantially higher adjusted mortality even when occurring after index hospitalization. We report for the first time that POAA was frequently associated with emergency visits or rehospitalization for arrhythmia, highlighting its clinical burden well beyond the perioperative period. Postoperative beta‐blocker use was associated with markedly less POAA, a novel finding suggesting a potential prophylactic role. Overall, these findings challenge the traditional view of POAA as benign and highlight the need for tailored, evidence‐based guidelines in this high‐risk population.

**Abbreviations**
AAD: Anti‐arrhythmic drug
AF: Atrial fibrillation
AFL: Atrial flutter
PAH: Pulmonary arterial hypertension
POAA: Postoperative atrial arrhythmia

## 1 Introduction

Lung transplantation is lifesaving for many patients with end stage pulmonary disease. However, lung transplant recipients continue to experience a high rate of postoperative complications, such as primary graft dysfunction, chronic lung allograft dysfunction, and increased risk of infection [1]. Postoperative atrial arrhythmia (POAA) is one of the most common complications, affecting approximately 20% [2] to 40% [3] of patients.

Despite its prevalence, the long‐term clinical implications of POAA are poorly understood. Most studies focus on early POAA during hospitalization, with limited data on late POAA occurring after hospital discharge [4]. While early POAA has been consistently associated with increased mortality [5, 6, 7, 8, 9], only one study has reported a similar association in late POAA [5]. Additionally, the risk factors for POAA in lung transplant recipients are not fully understood. Although some studies suggest associations with age [2], chronic obstructive pulmonary disease (COPD) [10], coronary artery disease (CAD) [8], male sex [8], and single versus double lung transplant [3, 11], findings remain inconsistent. Management of POAA in lung transplant patients is further complicated by a lack of evidence‐based guidelines, with most treatment decisions informed by guidelines for other thoracic [10] or general surgical [12] patients. Specifically, there are limited data on the outcomes after initiation of beta‐blockade or oral anticoagulation (OAC) in this population. Finally, evidence is sparse regarding the association between POAA and outcomes such as stroke [2, 8], myocardial infarction, or hospitalization for heart failure or arrhythmia care. Contemporary studies are especially needed to reflect the evolving lung transplant population, which now includes patients with COVID‐related lung disease accounting for 8.7% of all lung transplants in the United States from August 2020 to June 2022 [13].

Given the frequency and potential impact of POAA after lung transplant [14], it is critical that we gain a better understanding of its risk factors and clinical implications. This retrospective study aims to characterize the incidence, risk factors, outcomes, and treatment options for POAA in a large cohort. We hypothesize that POAA will be associated with significant morbidity and mortality, with an increased long‐term risk of conditions like stroke, MI, and new hospitalizations for atrial arrhythmia and heart failure.

## 2 Methods

### 2.1 Study Design and Population

We conducted a retrospective analysis of all patients enrolled in a lung transplant repository at Northwestern Memorial Hospital, which began enrolling patients in February 2021 (STU00212120). To ensure at least 6 months of follow up, we applied a surgical cut‐off date of October 23, 2024, yielding an initial cohort of 318 patients. Patients were excluded if they did not undergo lung transplant (*n* = 17), lacked index hospitalization records (*n* = 2), had preexisting atrial arrhythmia (*n* = 64), or underwent combined heart‐lung transplant (*n* = 2), leaving a final cohort of 233 patients. Follow‐up continued until death or April 23, 2025. This study was approved by the Northwestern IRB (STU00223074), with a waiver of informed consent. All procedures followed institutional and Helsinki guidelines. This study adhered to STROBE reporting standards.

### 2.2 Definitions

POAA was defined as atrial fibrillation or flutter occurring after lung transplant, confirmed by 12‐lead ECG or ambulatory rhythm monitoring and interpreted by a cardiologist. POAA was further classified as early (occurring during index hospitalization) or late (post‐discharge). All patients underwent a preoperative 12‐lead ECG, and continuous telemetry was used routinely during the postoperative hospitalization. After discharge, 12‐lead ECGs and ambulatory rhythm monitors were obtained at clinician discretion based on the clinical presentation. COPD and alpha‐1 antitrypsin deficiency were classified as obstructive lung disease. Interstitial pulmonary fibrosis, hypersensitivity pneumonitis, systemic autoimmune rheumatic disease‐associated interstitial lung disease (SARD‐ILD), and unspecified ILD were classified as restrictive lung disease. PAH was defined by mPAP > 21mmHg, PCWP < 15mmHg, and PVR > 2 Wood units.

### 2.3 Data Collection

Data on patient demographics, pretransplant comorbidities, clinical course, and outcomes were collected via manual chart review. For outcome analyses, POAA was only considered present if it occurred before the outcome of interest. Several steps were taken to minimize bias. To minimize observer bias, standardized protocols were used for data collection, and data collectors were not involved in patient care. To minimize misclassification bias, POAA required confirmation with 12‐lead ECG or ambulatory rhythm monitor. There were no missing data for variables included in the primary analysis. Missing data (detailed in Table S2) were limited to hemodynamic and echocardiographic variables and handled via complete case analysis. These data were used exclusively in a secondary mediation analysis. Hemodynamic data were taken from the last pretransplant right heart catheterization, and functional cardiac data from the echocardiogram obtained when the patient was listed for lung transplant.

### 2.4 Statistical Analysis

Analyses were conducted using R Version 4.5.0. Generative AI was used to suggest statistical tests and assist with coding. To determine risk factors associated with POAA, we initially performed univariate logistic regressions, and then built multivariable models using covariates with *p* < 0.25 or clinical relevance. Fisher's exact analysis was used to compare outcomes between patients with and without POAA. Univariate and multivariate Cox logistic regressions were used to determine variables associated with mortality. Finally, a multivariate time‐dependent Cox regression was used to determine whether postoperative beta‐blocker use was associated with POAA. Continuous data were reported as mean ± SD or median (IQR) as appropriate. *p* values for hypothesis‐driven analyses (e.g., association between POAA and mortality, postoperative beta‐blocker and POAA, and outcomes associated with POAA) were not adjusted for multiple comparisons due to the small number of prespecified tests. All other statistical tests were exploratory and hypothesis generating. The corresponding author has full access to all data and takes responsibility for its integrity.

## 3 Results

### 3.1 Descriptive Characteristics and Risk Factors for POAA

Table 1 summarizes characteristics of the 233 lung transplant patients. On average, patients with POAA were older, had more smoking pack years, were more likely to have CAD, and less likely to have hemodynamically defined PAH than patients without POAA. Indications for transplant included COVID‐related lung disease (*n* = 19; 8.2%), obstructive lung disease (*n* = 49; 21.0%), restrictive lung disease (*n* = 106; 45.5%), pulmonary hypertension (*n* = 16, 6.9%), and other (*n* = 42; 18.0%). Of the 69 patients who developed POAA, there were 37 (53.6%) patients that developed atrial fibrillation (AF), 17 (24.6%) patients that developed atrial flutter (AFL), and 15 (21.7%) patients that developed both. There were 18 (26.1%) patients that developed POAA (27.8% AF; 50% AFL; 22.2% both) after hospitalization, at a median time of 215.5 days after transplant (IQR 65.5–505.5 days). Incidence of early and late AF and AFL are summarized in Table S1.

**TABLE 1:** Descriptive characteristics and unadjusted risk factors for POAA.

Variable	Overall (*n* = 233, mean ± SD)	POAA (*n* = 69, mean ± SD)	No POAA (*n* = 164, mean ± SD)	OR (95% CI)	*p* value
Age	58.6 ± 12.8	62.5 ± 9.8	57.0 ± 13.6	1.04 (1.02, 1.07)	0.0036
Sex (female = ref)				1.68 (0.95, 3.02)	0.080
Male	128	44	84		
Female	105	25	80		
BMI	26.5 ± 4.7	26.4 ± 4.7	26.9 ± 4.7	1.02 (0.96, 1.09)	0.47
Type of Transplant (bilateral = ref)				1.51 (0.83, 2.71)	0.17
Unilateral (Left)	45	15	30		
Unilateral (Right)	31	12	19		
Bilateral	157	42	115		
Pack years	17.9 ± 24.7	23.9 ± 29.3	15.4 ± 22.1	1.01 (1.00, 1.02)	0.019
CAD	78	31	47	2.03 (1.13, 3.64)	0.017
CHF	60	23	37	1.72 (0.92, 3.18)	0.089
Hypertension	103	33	70	1.23 (0.70, 2.17)	0.47
PAH	78	12	66	0.31 (0.15, 0.61)	0.0011
COPD	78	27	51	1.42 (0.79, 2.55)	0.24
Sleep Apnea	54	18	36	1.25 (0.64, 2.39)	0.50
Anemia	26	10	16	1.57 (0.65, 3.61)	0.30
Diabetes	55	16	39	0.97 (0.49, 1.86)	0.92
Malignancy	43	14	29	1.18 (0.57, 2.38)	0.64
CKD	11	6	5	3.03 (0.88, 10.85)	0.076
Preoperative beta‐blockers	63	22	41	1.40 (0.75, 2.59)	0.28

Table 1 Baseline characteristics and unadjusted associations with postoperative atrial arrhythmia (POAA). Continuous variables are presented as mean ± standard deviation. Unadjusted logistic regression was used to compare characteristics between patients with and without POAA.

Abbreviations: BMI (body mass index), CAD (coronary artery disease), CHF (congestive heart failure), CKD (chronic kidney disease), COPD (chronic obstructive pulmonary disease), PAH (pulmonary arterial hypertension), POAA (postoperative atrial arrhythmia).

**FIGURE 1:**
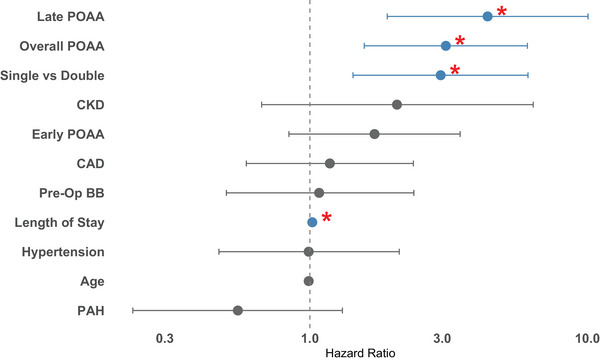
Adjusted risk factors for mortality. Multivariate regression model identifying factors associated with posttransplant mortality. The development of POAA was associated with increased mortality (HR 3.09, 95% CI 1.57–6.08, *p* = 0.0011), as were single lung transplant versus double (HR 2.93, 95% CI 1.41–6.07, *p* = 0.0039), and longer hospital length of stay (HR 1.02, 95% CI 1.01–1.03, *p* = 0.0000052). Abbreviations: CAD (coronary artery disease), CHF (congestive heart failure), CKD (chronic kidney disease), COPD (chronic obstructive pulmonary disease), PAH (pulmonary arterial hypertension).

Table 1 also shows which risk factors were associated with POAA in unadjusted univariate models. Age, number of pack years, and CAD were associated with more POAA, while pulmonary arterial hypertension (PAH) was found to have a protective association. All factors with a *p* value of < 0.25 on univariate analysis were included in a multivariate regression model (Figure 1). On multivariate analysis, a history of PAH was found to have a protective association against developing POAA (OR 0.31, 95% CI 0.14–0.62, *p* = 0.0015). The other factors did not have statistically significant associations (Table S2). Because there was a protective association between PAH and POAA, we performed a nonparametric bootstrapped mediation analysis with 5000 iterations. Several candidate mediators were assessed, including both hemodynamic (mPAP, PCWP, CO, PVR) and functional (RVSF, TR) variables. None of the mediators demonstrated a statistically significant mediation effect (Table S3), and the analyses could not be completed for the functional measures (RVSF, TR, RV size) because bootstrap iterations failed to converge.

We ran separate univariate and multivariate models for early and late POAA, and found that age (OR 1.04, 95% CI 1.01–1.08, *p* = 0.010) and PAH (OR 0.35, 95% CI 0.15–0.74, *p* = 0.0085) were both associated with early POAA in univariate models (Table S4). There were no significant predictors of early arrhythmia in our multivariate model, and there were no significant predictors of late POAA in univariate models (Tables S5 and S6). We also ran separate analyses for post‐operative AF and AFL, and found that age (OR 1.06, 95% CI 1.02–1.10, *p* = 0.0087) was associated with postoperative AF in univariate models. For AFL, PAH (OR 0.11, 95% CI 0.01–0.57, *p* = 0.036) was the only significant univariate predictor, and for mixed AF/AFL, recipient pack years was the only significant univariate predictor (OR 1.02, 95% CI 1.00–1.04, *p* = 0.038). In multivariate models, there were no significant predictors of AF, AFL or mixed AF/AFL (Tables S7–S10).

We attempted to perform a propensity match analysis to determine whether single or double lung transplant was more likely to be associated with POAA. However, a 1:2 nearest neighbor propensity match failed due to non‐overlap between propensity scores. A Kaplan–Meier analysis was performed, and there was no statistically significant difference in time to atrial arrhythmia between single and double lung transplant patients (HR 1.40, 95% CI 0.87–2.28, *p* = 0.17, Figure S1). The median length of stay was 17 days (IQR 11–31) in patients with POAA and 15 days (IQR 11–26) in those without POAA. The difference was not statistically significant (*p* = 0.14, Wilcoxon rank sum test).

### 3.2 Management of POAA and Outcomes Associated With POAA

Management strategies for the 69 patients who developed POAA are described in Table 2. Among the patients who developed POAA during hospitalization, only one patient was discharged on anticoagulation (CHA_2_DS_2_‐VASC 5, no history of stroke). 33 patients were started on anticoagulation during follow‐up. Anticoagulation for POAA was initiated at a median of 41 (IQR 8–173.25) days post‐transplant and was continued for an average of 17.3 ± 13.2 months. More patients were anticoagulated for DVT/PE (*n* = 25, 36.2%) than AF/AFL (*n* = 7, 10.1%). Prescription of anticoagulation by CHA_2_DS_2_‐VASC score is described in Table S11.

**TABLE 2:** Management strategies for POAA.

Management strategy	Overall POAA (*n* = 69)	Early POAA (*n* = 51)	Late POAA (*n* = 18)
Rate only	16	9	7
AAD only	9	8	1
Rate, AAD	23	18	5
Rate, DCCV	2	1	1
AAD, DCCV	3	3	0
Rate, AAD, DCCV	9	8	1
None	7	4	3

*Note:* Management strategies for post‐operative atrial arrhythmia (POAA), stratified by timing of arrhythmia onset.

Abbreviations: AAD (antiarrhythmic drug), DCCV (direct current cardioversion), POAA (postoperative atrial arrhythmia).

Table 3 compares the incidence of stroke, MI, and new hospitalization for AA or heart failure among patients that developed POAA and patients that did not develop POAA. We used Fisher's exact test because of the low incidence rates of all outcome variables. Of the patients that developed POAA, 14.5% (10/69) presented to the ED or were hospitalized for arrhythmia during follow‐up, compared to 0% of patients that did not develop POAA. Of these 10 patients, 3 had early POAA and 7 had late POAA. Two patients had a stroke after developing early POAA. Both patients were not on anticoagulation at the time. One patient had a CHA_2_DS_2_‐VASC score of 6, with stroke occurring 6 days after transplant and 2 days after POAA; and the other patient had a CHA_2_DS_2_‐VASC of 5, with stroke occurring 71 days after transplant and 50 days after POAA.

**TABLE 3:** Outcomes associated with POAA.

Outcome	Overall (*n* = 233)	POAA (*n* = 69)	No POAA (*n* = 164)	OR (95% CI) *p* value
Stroke				0.38 (0.08, 1.83) *p* = 0.26
< 30 d	6	1	5	
30 d–1 y	5	1	4	
1 y–3 y	3	0	3	
3 y–5 y	0	0	0	
> 5 y	0	0	0	
Total	14	2	12	
Follow‐up duration (d) (mean ± SD)	177 ± 256	39 ± 46	200 ± 262	
MI				1.20 (0.35, 4.12) *p* = 0.75
< 30 d	2	1	1	
30 d–1y	4	2	2	
1 y–3 y	6	1	5	
3 y–5 y	0	0	0	
> 5 y	0	0	0	
Total	12	4	8	
Follow‐up duration (d) (mean ± SD)	480 ± 392	299 ± 414	609 ± 347	
AA Hospitalization				∞ (2.2, ∞) *p* < 0.0001
< 30 d	4	4	0	
30 d–1 y	4	4	0	
1 y–3 y	1	1	0	
3 y–5 y	1	1	0	
> 5 y	0	0	0	
Total	10	10	0	
Follow‐up duration (d) (mean ± SD)	336 ± 523	311 ± 495	N/A	
HF Hospitalization				1.21 (0.49, 2.98) *p* = 0.65
< 30 d	0	0	0	
30 d–1 y	14	4	10	
1 y–3 y	6	2	4	
3 y–5 y	4	2	2	
> 5 y	0	0	0	
Total	24	8	16	
Follow‐up duration (d) (mean ± SD)	439 ± 426	425 ± 385	448 ± 426	

*Note:* Clinical outcomes associated with post‐operative atrial arrhythmia (POAA). Events are stratified by POAA status and time since transplant (< 30 days, 30 days–1 year, 1–3 years, 3–5 years, > 5 years). Fisher's exact test was used to compare outcomes between patients with and without POAA.

^§^Abbreviations: AA (atrial arrhythmia), HF (heart failure), MI (myocardial infarction), POAA (post‐operative atrial arrhythmia).

### 3.3 Predictors of Mortality

Table S12 describes which variables are associated with mortality in univariate Cox regression models. Single lung transplant, length of hospitalization, CKD, and POAA (overall, early, and late) were all found to be significantly associated with increased mortality. PAH had a protective association against mortality.

All factors with *p* < 0.25 in our unadjusted model were included in a multivariate regression analysis (Figure S2). Significant predictors of mortality in the adjusted model included overall POAA (HR 3.09, 95% CI 1.57–6.08, *p* = 0.0011), single versus double lung transplant (HR 2.93, 95% CI 1.41–6.07, *p* = 0.0039), and length of hospital stay (HR 1.02, 95% CI 1.01–1.03, *p* = 0.0000052). The multivariate regression analysis was re‐run with early and late POAA, and late POAA was also found to be a significant predictor of mortality (HR 4.37, 95% CI 1.90–10.06, *p* = 0.00051). There was no significant association between early POAA and mortality (Table S13).

### 3.4 Effects of Postoperative Beta‐Blockers on POAA Risk

A total of 141 patients received postoperative beta‐blockers after lung transplant surgery, including 82 who were beta‐blocker naïve prior to transplant. These beta‐blockers were started an average of 8 ± 6 days after transplant. We ran a time‐dependent multivariate Cox regression analysis to see whether postoperative beta‐blocker use had a protective association against POAA (Table S14). Postoperative beta‐blocker use was associated with a significantly reduced hazard of developing arrhythmia (HR 0.27, 95% CI 0.09–0.80, *p* = 0.018).

## 4 Discussion

In this single‐center retrospective cohort study of 233 patients, the incidence of postoperative atrial arrhythmia was 29.6%, with the majority of arrhythmia being AF. Postoperative beta‐blockers and a history of preoperative PAH were associated with less POAA in adjusted models. Overall and late (but not early) POAA were both found to be associated with increased mortality in an adjusted model, and 14.5% of patients with POAA presented to the ED or were hospitalized for arrhythmia during follow‐up. Management strategies for POAA tended to favor rate control, and only 7/69 patients with POAA were prescribed anticoagulation during follow‐up. There were two POAA patients not on anticoagulation who suffered an ischemic stroke.

Although POAA has traditionally been considered a self‐limiting and benign phenomenon, our study adds to a growing body of evidence that challenges this view. In our cohort, POAA after lung transplant was common, associated with substantially increased mortality, and linked to frequent arrhythmia‐related ED or hospital encounters. Our study is the first to quantify such encounters in this population, revealing that 14.5% of patients with POAA experienced these events. Moreover, we are among the few studies [5] to report the frequency and outcomes associated with POAA developing after the index hospitalization. While these observations do not establish causality, they suggest a possible role for long‐term rhythm monitoring to better define arrhythmia burden and associated risks. Because patients are not routinely monitored for arrhythmia after lung transplant, it is likely that our study did not capture cases of subclinical arrhythmia. Overall, our findings suggest that POAA may carry greater clinical significance than previously recognized, warranting further study in the lung transplant population.

Notably, our study is the first to demonstrate an association between postoperative beta‐blocker uses and early (in‐hospital) POAA after lung transplant. While another study reports no significant difference in POAA between patients who were or were not taking beta‐blockers at time of transplant [7], their analysis uses an unadjusted Chi‐square test, and we believe that this study's adjusted time‐dependent Cox regression better accounts for covariates and temporal dependence. These findings should be interpreted with caution, as clinicians likely prescribed beta blockers preferentially to more stable patients, introducing potential selection bias. While our regression model adjusts for covariates, unmeasured confounding remains possible, and our results should be considered hypothesis‐generating. Nevertheless, our observed association (HR 0.27, 95% CI 0.09–0.80, *p* = 0.018) is compelling, and supports the need for larger randomized trials to assess the role of prophylactic beta‐blockers in the lung transplant population.

While prophylactic beta‐blocker use is well studied in cardiac surgery patients [15, 16] and strongly endorsed by the 2020 ESC guidelines (Class 1a) [17], its role in thoracic surgery patients remains controversial. The most recent AATS [18] guidelines discourage beta‐blocker initiation in beta‐blocker naïve patients because of increased stroke and mortality risk demonstrated in the POISE trial [19]. However, that trial included a heterogenous noncardiac surgical population, limiting its applicability. In contrast, three RCTs [20, 21, 22] and two more recent large meta‐analyses [23, 24] conducted specifically in thoracic surgery populations demonstrate that low‐dose beta‐blockers are effective and well tolerated. These findings have not been reflected in updated recommendations. We argue that data from general noncardiac surgery patients cannot be extrapolated to thoracic surgery patients, who experience a substantially higher incidence of POAA. Overall, our findings highlight a potentially protective role for beta‐blockers in lung transplant recipients and emphasize the need for more transplant‐specific RCTs and guidelines.

Beyond beta‐blockers, the overall management of POAA in lung transplant recipients remains challenging. The use of amiodarone, for example, has been controversial: some studies associate it with increased pulmonary toxicity [25] and mortality [8], while other studies advocate for it as a first‐line agent for treating POAA after lung transplant [10]. Another important consideration is the initiation of anticoagulation. While current AF guidelines recommend utilizing the CHA_2_DS_2_‐VASc score, application of these recommendations to lung transplant recipients is complicated by frequent bronchoscopies and postoperative bleeding risk. Therefore, only a small proportion of patients in our cohort with POAA were anticoagulated specifically for AF/AFL, and two ischemic strokes occurred in patients who were not receiving anticoagulation at the time of the event. The occurrence of strokes in non‐anticoagulated patients highlights the need for clearer guidance regarding anticoagulation thresholds and duration, as well as studies to evaluate thromboembolic and bleeding risks in this unique group.

Finally, it was surprising that a preoperative history of PAH had a negative association with POAA, even while correcting for age. We suspect that for patients with PAH, lung transplant may relieve pressure on the right atrium and ventricle, leading to cardiac remodeling and less susceptibility to POAA. This interpretation is supported by several studies that demonstrate reverse remodeling of the RV following lung transplant in this population [26, 27, 28]. Our finding is similar to that of Chaikriangkrai et al. [29], who found that mPAP was inversely associated with POAA. More data are needed to confirm this finding and determine how it may affect the management of lung transplant patients with PAH.

This study has several limitations. First, as mentioned previously, the true incidence of POAA may have been underestimated. POAA was classified only when documented on a 12‐lead ECG or ambulatory monitor, and outpatient ECGs at our center are not routinely performed at every follow‐up visit. After discharge, ECGs and ambulatory monitors were obtained only when prompted by symptoms or other clinical concerns. As a result, paroxysmal or subclinical atrial arrhythmias occurring outside of these recordings may not have been captured, and the true burden and longitudinal course of POAA was unable to be determined. Second, our study is a retrospective, single‐center study, which may limit generalizability and introduce bias related to documentation and clinical practice patterns. Third, the study was underpowered to detect associations with less frequent clinical outcomes such as heart failure hospitalization, stroke, or mortality. Fourth, the management of atrial arrhythmias was not standardized and was subject to variability based on physician judgment and individual patient factors, which may have influenced outcomes. Finally, patients who developed POAA were not routinely evaluated for other cardiac comorbidities at time of POAA diagnosis. Due to the retrospective nature of our study, we were unable to determine whether progression of underlying cardiovascular disease contributed to POAA risk.

In conclusion, in this retrospective study of lung transplant patients, POAA was common, associated with higher mortality, and linked to frequent arrhythmia‐related ED visits and hospitalizations. Our novel findings challenge the traditional view of POAA as a benign and self‐limited condition, particularly given the late onset of arrhythmia in some patients and its association with adverse outcomes. Postoperative beta‐blocker therapy was associated with a lower incidence of in‐hospital POAA, which is also a novel observation in this population. Future studies should aim to assess true arrhythmia burden in this population, evaluate long‐term outcomes, and utilize standardized treatment protocols to better understand POAA management strategies.

## Conflicts of Interest

The authors declare the following financial interests/personal relationships which may be considered as potential competing interests: Dr. Graham Peigh reports consulting fees from Philips and Medtronic. Dr. Mohamed Al‐Kazaz reports research grant support from Kiniksa Pharmaceuticals, Ventyx BioSciences, and Cardiol Therapeutics; speaking honoraria from Kiniksa Pharmaceuticals, and consulting fees from Edwards Lifesciences. Other authors declare that they have no known competing financial interests or personal relationships that could have appeared to influence the work reported in this paper.

## Supporting information



Supporting file 1: ctr70457‐sup‐0001‐figureS1.pdf

Table S1: Incidence of Early and Late Post‐Operative AF and AFL.
Table S2: Adjusted Risk Factors for POAA.
Table S3: Mediators of PAH and POAA.
Table S4: Unadjusted Risk Factors for Early POAA.
Table S5: Adjusted Risk Factors for Early POAA.
Table S6: Unadjusted Risk Factors for Late POAA.
Table S7: Unadjusted Risk Factors for Post‐Operative AF.
Table S8: Adjusted Risk Factors for Post‐Operative AF.
Table S9: Unadjusted Risk Factors for Post‐Operative AFL.
Table S10: Unadjusted Risk Factors for Post‐Operative Mixed AF/AFL.
Table S11: Anticoagulation Prescription by CHA_2_DS_2_‐VASC Score.
Table S12: Unadjusted predictors of mortality.
Table S13: Adjusted predictors of mortality.
Table S14: Adjusted effect of post‐operative beta blockers on POAA risk.
Figure S1: Arrhythmia‐free survival by single vs. double lung transplant.
Figure S2: Adjusted risk factors for POAA.

## Data Availability

The data that support the findings of this study are available from the corresponding author upon reasonable request.
