# Supplementary figures and images for: Postoperative Atrial Arrhythmias After Lung Transplantation: A Single Center Analysis of Risk Factors, Management, and Outcomes
Source: Clin Transplant. 2026 Jan 30;40(2):e70457. doi: 10.1111/ctr.70457 (PMC12857598; doi:10.1111/ctr.70457)

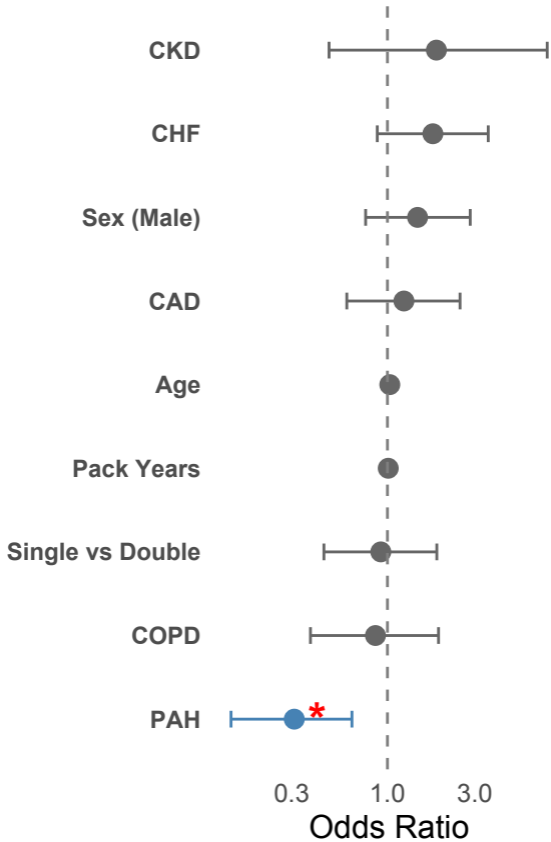

Supplement: Supplementary file 1 — Supporting file 1: ctr70457‐sup‐0001‐figureS1.pdf [file CTR-40-e70457-s001.pdf]
