# Supplementary material for: Postoperative Atrial Arrhythmias After Lung Transplantation: A Single Center Analysis of Risk Factors, Management, and Outcomes
Source: Clin Transplant. 2026 Jan 30;40(2):e70457. doi: 10.1111/ctr.70457 (PMC12857598; doi:10.1111/ctr.70457)
Supplement: Supplementary file 2 — Table S1: Incidence of Early and Late Post‐Operative AF and AFL. Table S2: Adjusted Risk Factors for POAA. Table S3: Mediators of PAH and POAA. Table S4: Unadjusted Risk Factors for Early POAA. Table S5: Adjusted Risk Factors for Early POAA. Table S6: Unadjusted Risk Factors for Late POAA. Table S7: Unadjusted Risk Factors for Post‐Operative AF. Table S8: Adjusted Risk Factors for Post‐Operative AF. Table S9: Unadjusted Risk Factors for Post‐Operative AFL. Table S10: Unadjusted Risk Factors for Post‐Operative Mixed AF/AFL. Table S11: Anticoagulation Prescription by CHA2DS2‐VASC Score. Table S12: Unadjusted predictors of mortality. Table S13: Adjusted predictors of mortality. Table S14: Adjusted effect of post‐operative beta blockers on POAA risk. Figure S1: Arrhythmia‐free survival by single vs. double lung transplant. Figure S2: Adjusted risk factors for POAA. [file CTR-40-e70457-s002.docx]

**SUPPLEMENTARY MATERIAL**

**Table S1: Incidence of Early and Late Post-Operative AF and AFL**

| **Arrhythmia Type** | **Total and Incidence (% of cohort, n = 233)** | **Early (During Index Hospitalization)** | **Late (After Discharge)** |
| --- | --- | --- | --- |
| **Total POAA**† | 69 (29.6%) | 51 (21.9%) | 18 (7.7%) |
| **AF** | 37 (15.9%) | 32 (13.7%) | 5 (2.1%) |
| **AFL** | 17 (7.3%) | 8 (3.4%) | 9 (3.9%) |
| **Mixed AF/AFL** | 15 (6.4%) | 11 (4.7%) | 4 (1.7%) |

**Table S1:** Incidence and percentages of post-operative atrial arrhythmias following lung transplantation, stratified by type of arrhythmia and timing of onset.

†Abbreviations: POAA (post-operative atrial arrhythmia), AF (atrial fibrillation), AFL (atrial flutter)

**Table S2: Adjusted Risk Factors for POAA**

| **Variable** | **OR (95% CI)** | **p-value** |
| --- | --- | --- |
| **Age** | 1.03 (1.00–1.07) | 0.053 |
| **Sex (male)** | 1.50 (0.78–2.90) | 0.23 |
| **Single vs Double** | 0.95 (0.47–1.90) | 0.88 |
| **Pack Years** | 1.01 (0.99–1.02) | 0.35 |
| **CAD**‡ | 1.18 (0.58–2.38) | 0.64 |
| **CHF** | 1.77 (0.88–3.55) | 0.11 |
| **PAH** | 0.31 (0.14–0.62) | 0.0015 |
| **COPD** | 0.89 (0.39–1.97) | 0.77 |
| **CKD** | 1.83 (0.47–7.38) | 0.38 |

**Table S2:** Adjusted associations between clinical variables and risk of post-operative atrial arrhythmia (POAA), as determined by multivariate logistic regression.

‡Abbreviations: CAD (coronary artery disease), CHF (congestive heart failure), PAH (pulmonary arterial hypertension), COPD (chronic obstructive pulmonary disease), CKD (chronic kidney disease)

**Table S3: Mediators of PAH and POAA**

| **Mediator** | **a** | **b** | **Indirect Effect (95% CI)** | **p-value** | **Missing** |
| --- | --- | --- | --- | --- | --- |
| **mPAP**§ | 6.18 | 0.02 | 0.10 (-0.10 to 0.33) | 0.28 | 18 |
| **PCWP** | -3.71 | 0.04 | -0.15 (-0.41 to 0.05) | 0.16 | 21 |
| **CO (thermodilution)** | -0.15 | 0.07 | -0.009 (-0.09 to 0.05) | 0.78 | 29 |
| **CO (Fick)** | -0.42 | -0.05 | 0.03 (-0.06 to 0.16) | 0.63 | 44 |
| **CI (thermodilution)** | -0.12 | -0.19 | 0.02 (-0.04 to 0.12) | 0.57 | 29 |
| **CI (Fick)** | -0.22 | -0.36 | 0.08 (-0.02 to 0.28) | 0.18 | 44 |
| **PVR** | 1.96 | 0.002 | 0.0001 (-0.37 to 0.28) | 0.93 | 22 |
| **RA** | -1.32 | 0.02 | -0.01 (-0.13 to 0.07) | 0.86 | 21 |
| **LVEF** | -2.00 | -0.02 | 0.03 (-0.08 to 0.15) | 0.53 | 15 |

**Table S3:** Mediators of the association between pulmonary arterial hypertension (PAH) and post-operative atrial arrhythmia (POAA), as determined by nonparametric bootstrapped mediation analysis with 5,000 iterations. Coefficients **a** and **b** reflect the paths from PAH to the mediator and from the mediator to POAA, respectively.

§Abbreviations: mPAP (mean pulmonary artery pressure), PCWP (mean pulmonary capillary wedge pressure), CO (cardiac output), CI (cardiac index), PVR (pulmonary vascular resistance), RA (mean right atrial pressure), LVEF (left ventricular ejection fraction)

**Table S4: Unadjusted Risk Factors for Early POAA**

| **Variable** | **OR (95% CI)** | **p-value** |
| --- | --- | --- |
| **Age** | 1.04 (1.01–1.08) | 0.010 |
| **Sex (Male)** | 1.87 (0.99–3.66) | 0.059 |
| **BMI**¶ | 1.01 (0.95–1.09) | 0.66 |
| **Single vs Double** | 1.30 (0.67–2.47) | 0.42 |
| **Pack Years** | 1.01 (1.00–1.02) | 0.12 |
| **CAD** | 1.90 (1.00–3.58) | 0.049 |
| **CHF** | 1.82 (0.92–3.53) | 0.080 |
| **HTN** | 1.57 (0.84–2.94) | 0.16 |
| **PAH** | 0.35 (0.15–0.74) | 0.0085 |
| **COPD** | 1.38 (0.72–2.61) | 0.33 |
| **Anemia** | 1.70 (0.66–4.05) | 0.25 |
| **OSA** | 1.18 (0.56–2.37) | 0.66 |
| **Diabetes** | 0.86 (0.39–1.78) | 0.70 |
| **Malignancy** | 1.29 (0.58–2.72) | 0.52 |
| **CKD** | 3.19 (0.88–11.04) | 0.065 |
| **Beta Blockers Prior to Admission** | 1.66 (0.84–3.21) | 0.14 |

**Table S4:** Unadjusted associations between clinical variables and risk of early post-operative atrial arrhythmia (POAA), as determined by univariate logistic regression.

¶Abbreviations: BMI (body mass index), CAD (coronary artery pressure), CHF (congestive heart failure), HTN (hypertension), PAH (pulmonary arterial hypertension), COPD (chronic obstructive pulmonary disease), OSA (obstructive sleep apnea), CKD (chronic kidney disease)

**Table S5: Adjusted Risk Factors for Early POAA**

| **Variable** | **OR (95% CI)** | **p-value** |
| --- | --- | --- |
| **Age** | 1.04 (1.00–1.08) | 0.044 |
| **Sex (Male)** | 1.96 (0.93–4.30) | 0.083 |
| **Pack Years** | 1.00 (0.99–1.01) | 0.83 |
| **CAD**† | 0.94 (0.43–2.02) | 0.88 |
| **CHF** | 1.86 (0.87–3.92) | 0.10 |
| **HTN** | 1.17 (0.55–2.46) | 0.68 |
| **PAH** | 0.35 (0.15–0.77) | 0.012 |
| **Anemia** | 1.97 (0.68–5.42) | 0.20 |
| **CKD** | 1.80 (0.44–7.13) | 0.40 |
| **Beta Blockers Prior to Admission** | 1.36 (0.62–2.93) | 0.44 |

**Table S5:** Adjusted associations between clinical variables and risk of early post-operative atrial arrhythmia (POAA), as determined by multivariate logistic regression.

†Abbreviations: CAD (coronary artery disease), CHF (congestive heart failure), HTN (hypertension), PAH (pulmonary arterial hypertension), CKD (chronic kidney disease)

**Table S6: Unadjusted Risk Factors for Late POAA**

| **Variable** | **OR (95% CI)** | **p-value** |
| --- | --- | --- |
| **Age** | 1.02 (0.98–1.08) | 0.30 |
| **Sex (Male)** | 1.03 (0.39–2.79) | 0.96 |
| **BMI**‡ | 1.03 (0.93–1.15) | 0.57 |
| **Single vs Double** | 1.73 (0.63–4.58) | 0.27 |
| **Pack Years** | 1.01 (1.00–1.03) | 0.11 |
| **CAD** | 1.66 (0.61–4.38) | 0.31 |
| **CHF** | 1.12 (0.35–3.12) | 0.84 |
| **HTN** | 0.61 (0.21–1.63) | 0.34 |
| **PAH** | 0.37 (0.08–1.18) | 0.13 |
| **COPD** | 1.29 (0.46–3.42) | 0.61 |
| **Anemia** | 0.99 (0.15–3.79) | 0.99 |
| **OSA** | 1.30 (0.40–3.64) | 0.63 |
| **Diabetes** | 1.27 (0.39–3.55) | 0.66 |
| **Malignancy** | 0.88 (0.20–2.81) | 0.84 |
| **CKD** | 1.21 (0.06–6.88) | 0.86 |
| **Beta Blockers Prior to Admission** | 0.76 (0.21–2.21) | 0.63 |

**Table S6:** Unadjusted associations between clinical variables and risk of late post-operative atrial arrhythmia (POAA), as determined by univariate logistic regression.

‡Abbreviations: BMI (body mass index), CAD (coronary artery pressure), CHF (congestive heart failure), HTN (hypertension), PAH (pulmonary arterial hypertension), COPD (chronic obstructive pulmonary disease), OSA (obstructive sleep apnea), CKD (chronic kidney disease)

**Table S7: Unadjusted Risk Factors for Post-Operative AF**

| **Variable** | **OR (95% CI)** | **p-value** |
| --- | --- | --- |
| **Age** | 1.06 (1.02–1.10) | 0.0087 |
| **Sex (Male)** | 1.63 (0.80–3.48) | 0.19 |
| **BMI**§ | 1.02 (0.95–1.10) | 0.62 |
| **Single vs Double** | 1.97 (0.96–4.04) | 0.062 |
| **Pack Years** | 1.00 (0.99–1.02) | 0.70 |
| **CAD** | 1.88 (0.91–3.84) | 0.083 |
| **CHF** | 1.27 (0.57–2.70) | 0.55 |
| **HTN** | 1.82 (0.90–3.76) | 0.10 |
| **PAH** | 0.59 (0.25–1.28) | 0.20 |
| **COPD** | 0.95 (0.43–1.97) | 0.88 |
| **Anemia** | 0.66 (0.15–2.05) | 0.52 |
| **OSA** | 1.28 (0.55–2.78) | 0.55 |
| **Diabetes** | 0.58 (0.21–1.39) | 0.25 |
| **Malignancy** | 1.53 (0.63–3.44) | 0.32 |
| **CKD** | 3.27 (0.82–11.47) | 0.070 |
| **Beta Blockers Prior to Admission** | 1.36 (0.62–2.87) | 0.42 |

**Table S7:** Unadjusted associations between clinical variables and risk of post-operative atrial fibrillation (AF), as determined by univariate logistic regression.

§Abbreviations: BMI (body mass index), CAD (coronary artery pressure), CHF (congestive heart failure), HTN (hypertension), PAH (pulmonary arterial hypertension), COPD (chronic obstructive pulmonary disease), OSA (obstructive sleep apnea), CKD (chronic kidney disease)

**Table S8: Adjusted Risk Factors for Post-Operative AF**

| **Variable** | **OR (95% CI)** | **p-value** |
| --- | --- | --- |
| **Age** | 1.04 (1.00–1.10) | 0.07 |
| **Sex** | 1.33 (0.61–2.98) | 0.48 |
| **Single vs Double** | 1.22 (0.53–2.75) | 0.64 |
| **CAD**¶ | 1.08 (0.47–2.44) | 0.86 |
| **HTN** | 1.38 (0.65–2.97) | 0.40 |
| **PAH** | 0.62 (0.26–1.40) | 0.27 |
| **CKD** | 2.78 (0.66–10.58) | 0.14 |

**Table S8:** Adjusted associations between clinical variables and risk of post-operative atrial fibrillation (AF), as determined by multivariate logistic regression.

¶Abbreviations: CAD (coronary artery disease), HTN (hypertension), PAH (pulmonary arterial hypertension), CKD (chronic kidney disease)

**Table S9: Unadjusted Risk Factors for Post-Operative AFL**

| **Variable** | **OR (95% CI)** | **p-value** |
| --- | --- | --- |
| **Age** | 1.02 (0.98–1.08) | 0.35 |
| **Sex** | 0.71 (0.26–1.93) | 0.50 |
| **BMI**† | 0.97 (0.87–1.08) | 0.53 |
| **Single vs Double** | 0.85 (0.26–2.39) | 0.77 |
| **Pack Years** | 1.01 (1.00–1.03) | 0.11 |
| **CAD** | 1.09 (0.36–2.99) | 0.87 |
| **CHF** | 1.64 (0.54–4.52) | 0.35 |
| **HTN** | 0.36 (0.10–1.07) | 0.09 |
| **PAH** | 0.11 (0.01–0.57) | 0.036 |
| **COPD** | 1.43 (0.50–3.88) | 0.49 |
| **Anemia** | 2.71 (0.72–8.46) | 0.10 |
| **OSA** | 0.69 (0.16–2.23) | 0.58 |
| **Diabetes** | 1.38 (0.42–3.93) | 0.56 |
| **Malignancy** | 0.94 (0.21–3.06) | 0.93 |
| **CKD** | 3.07 (0.44–13.31) | 0.18 |
| **Beta Blockers Prior to Admission** | 1.14 (0.35–3.21) | 0.82 |

**Table S9:** Unadjusted associations between clinical variables and risk of post-operative atrial flutter (AFL), as determined by univariate logistic regression.

†Abbreviations: BMI (body mass index), CAD (coronary artery pressure), CHF (congestive heart failure), HTN (hypertension), PAH (pulmonary arterial hypertension), COPD (chronic obstructive pulmonary disease), OSA (obstructive sleep apnea), CKD (chronic kidney disease)

**Table S10: Unadjusted Risk Factors for Post-Operative Mixed AF/AFL**

| **Variable** | **OR (95% CI)** | **p-value** |
| --- | --- | --- |
| **Age** | 1.01 (0.97–1.07) | 0.59 |
| **Sex** | 3.52 (1.08–15.75) | 0.057 |
| **BMI**‡ | 1.08 (0.96–1.22) | 0.21 |
| **Single vs Double** | 1.04 (0.31–3.03) | 0.95 |
| **Pack Years** | 1.02 (1.00–1.04) | 0.038 |
| **CAD** | 2.42 (0.84–7.15) | 0.10 |
| **CHF** | 2.02 (0.65–5.88) | 0.20 |
| **HTN** | 1.48 (0.51–4.36) | 0.46 |
| **PAH** | 0.29 (0.04–1.08) | 0.11 |
| **COPD** | 2.42 (0.84–7.15) | 0.10 |
| **Anemia** | 2.12 (0.46–7.28) | 0.27 |
| **OSA** | 1.72 (0.52–5.10) | 0.34 |
| **Diabetes** | 1.68 (0.50–4.96) | 0.36 |
| **Malignancy** | 0.66 (0.10–2.53) | 0.60 |
| **CKD** | 0.00 (NA–3.48e+31) | 0.99 |
| **Beta Blockers Prior to Admission** | 1.38 (0.42–4.06) | 0.57 |

**Table S10:** Unadjusted associations between clinical variables and risk of post-operative mixed atrial fibrillation and atrial flutter (AF/AFL), as determined by univariate logistic regression.

‡Abbreviations: BMI (body mass index), CAD (coronary artery pressure), CHF (congestive heart failure), HTN (hypertension), PAH (pulmonary arterial hypertension), COPD (chronic obstructive pulmonary disease), OSA (obstructive sleep apnea), CKD (chronic kidney disease)

**Table S11: Anticoagulation Prescription by CHA_2_DS_2_-VASC Score**

| **CHA_2_DS_2_-VASC** | **Number of Patients** | **Number on AC** |
| --- | --- | --- |
| 0 | 2 | 0 |
| 1 | 16 | 9 |
| 2 | 17 | 8 |
| 3 | 14 | 8 |
| 4 | 10 | 4 |
| 5 | 4 | 2 |
| 6 | 5 | 2 |
| 7 | 1 | 0 |

**Table S11: Anticoagulation Prescription by CHA₂DS₂-VASc Score.** Number of patients prescribed anticoagulation (AC) following post-operative atrial arrhythmia, stratified by CHA₂DS₂-VASc score.

**Table S12: Unadjusted predictors of mortality**

| **Variable** | **HR (95% CI)** | **p-value** |
| --- | --- | --- |
| **Age** | 1.02 (0.99–1.05) | 0.15 |
| **Sex (Male)** | 0.87 (0.48–1.59) | 0.65 |
| **BMI**§ | 0.97 (0.91–1.04) | 0.42 |
| **Single vs Double** | 2.84 (1.55–5.19) | 0.00069 |
| **Pack Years** | 1.00 (0.98–1.01) | 0.50 |
| **Length of Hospitalization** | 1.03 (1.02–1.03) | 0.000000041 |
| **CAD** | 1.66 (0.90–3.06) | 0.10 |
| **CHF** | 1.42 (0.75–2.67) | 0.28 |
| **DVT/PE** | 1.14 (0.48–2.70) | 0.77 |
| **HTN** | 1.44 (0.79–2.64) | 0.23 |
| **PAH** | 0.39 (0.18–0.84) | 0.017 |
| **COPD** | 1.39 (0.75–2.56) | 0.30 |
| **Anemia** | 0.59 (0.18–1.90) | 0.37 |
| **Sleep Apnea** | 0.66 (0.29–1.50) | 0.32 |
| **Diabetes** | 1.41 (0.72–2.77) | 0.31 |
| **Malignancy** | 1.20 (0.53–2.70) | 0.67 |
| **CKD** | 3.13 (1.11–8.85) | 0.031 |
| **Pre-Op Beta Blocker** | 1.60 (0.84–3.05) | 0.16 |
| **POAA (Overall)** | 4.06 (2.21–7.46) | 0.0000063 |
| **POAA (Early)** | 2.92 (1.57–5.41) | 0.00067 |
| **POAA (Late)** | 3.01 (1.39–6.53) | 0.0053 |

**Table S12:** Unadjusted predictors of mortality, as determined by univariate Cox regression.

§Abbreviations: BMI (body mass index), CAD (coronary artery pressure), CHF (congestive heart failure), DVT/PE (deep venous thrombosis/pulmonary embolism), HTN (hypertension), PAH (pulmonary arterial hypertension), COPD (chronic obstructive pulmonary disease), OSA (obstructive sleep apnea), CKD (chronic kidney disease), POAA (post-operative atrial arrhythmia)

**Table S13: Adjusted predictors of mortality**

| **Variable** | **HR (95% CI)** | **p-value** |
| --- | --- | --- |
| **Age** | 0.99 (0.96 – 1.02) | 0.46 |
| **Length of Hospitalization** | 1.02 (1.01 – 1.03) | 0.0000052 |
| **Single vs Double** | 2.93 (1.41 – 6.07) | 0.0039 |
| **CAD**¶ | 1.22 (0.61 – 2.43) | 0.58 |
| **HTN** | 0.95 (0.45 – 2.00) | 0.90 |
| **PAH** | 0.56 (0.24 – 1.33) | 0.19 |
| **CKD** | 2.07 (0.67 – 6.42) | 0.21 |
| **Beta Blockers Prior to Admission** | 1.12 (0.51 – 2.44) | 0.78 |
| **Overall POAA** | 3.09 (1.57 – 6.08) | 0.0011 |
| **Early POAA** | 1.71 (0.84 – 3.48) | 0.14 |
| **Late POAA** | 4.37 (1.90 – 10.06) | 0.00051 |

**Table S13:** Adjusted predictors of mortality, as determined by multivariate Cox regression.

¶Abbreviations: CAD (coronary artery pressure), HTN (hypertension), PAH (pulmonary arterial hypertension), CKD (chronic kidney disease), POAA (post-operative atrial arrhythmia)

**Table S14: Adjusted effect of post-operative beta blockers on POAA risk**

| **Variable** | **HR (95% CI)** | **p-value** |
| --- | --- | --- |
| **Post-Operative Beta Blocker** | 0.27 (0.093–0.80) | 0.018 |
| **Beta Blockers Prior to Admission** | 1.32 (0.42–4.15) | 0.63 |
| **Age** | 1.05 (1.00–1.09) | 0.051 |
| **Sex (Male)** | 2.84 (1.09–7.44) | 0.033 |
| **Pack Years** | 1.00 (0.99–1.02) | 0.68 |
| **CAD**† | 0.76 (0.28–2.10) | 0.60 |
| **CHF** | 1.57 (0.53–4.65) | 0.42 |
| **HTN** | 0.73 (0.28–1.91) | 0.52 |
| **PAH** | 0.45 (0.15–1.40) | 0.17 |
| **Anemia** | 1.53 (0.46–5.10) | 0.49 |
| **CKD** | 1.16 (0.17–7.81) | 0.88 |

**Table S14.** A time-adjusted multivariable Cox regression model was used to evaluate the effect of post-operative beta blocker use on post-operative atrial arrhythmia (POAA) risk, adjusting for demographic and clinical covariates.

†Abbreviations: CAD (coronary artery disease), CHF (congestive heart failure), HTN (hypertension), PAH (pulmonary arterial hypertension), CKD (chronic kidney disease)

**Figure S1: Arrhythmia-free survival by single vs. double lung transplant**

**Figure S1:** Kaplan-Meier curve stratifying arrhythmia-free survival by single vs. double lung transplant. There was no statistically significant difference in time to atrial arrhythmia between single and double lung transplant patients (HR 1.40, 95% CI 0.87–2.28, p = 0.17).

**Figure S2: Adjusted risk factors for POAA**

**Figure S2:** Multivariate regression model identifying risk factors for POAA. A preoperative history of PAH was found to have a protective association against POAA (OR 0.31, 95% CI 0.14–0.62, p = 0.0015).

Abbreviations: POAA (post-operative atrial arrhythmia), CKD (chronic kidney disease), CAD (coronary artery disease), BB (beta blocker), PAH (pulmonary arterial hypertension).
